# Real-world chemistry lab image dataset for equipment recognition across 25 apparatus categories

**DOI:** 10.1038/s41597-025-05952-3

**Published:** 2025-11-05

**Authors:** Md Sakhawat Hossain, Md Sadman Haque, Md Mostafizur Rahman, Md Mosaddik Mashrafi Mousum, Zobaer Ibn Razzaque, Robiul Awoul Robin, Raiyan Rahman, Jannatun Noor

**Affiliations:** 1 Department of Computer Science and Engineering, United International University, Dhaka, Bangladesh; 2 School of Computing, University of Portsmouth, Portsmouth, United Kingdom

**Keywords:** Chemical education, Research data

## Abstract

In modern laboratories, automation and safety rely heavily on accurately detecting and identifying laboratory equipment. To address this need, we introduce a comprehensive and well-curated dataset designed to detect 25 commonly used chemistry lab apparatuses. The dataset comprises 4,599 JPG-format images captured under diverse real-world conditions, including varying lighting, backgrounds, angles, overlaps, and distances - factors that enhance the robustness and generalizability of model training. It is split into training (70%), validation (20%), and testing (10%) subsets. This resource is particularly valuable for developing laboratory automation systems, with potential applications in safety monitoring, inventory management, and real-time tracking of lab tools. We evaluated the dataset using seven state-of-the-art object detection models, all achieving impressive performance with mAP@50 scores exceeding 0.9: RF-DETR (0.992), YOLOv11 (0.987), YOLOv9 (0.986), YOLOv5 (0.985), YOLOv8 (0.983), YOLOv7 (0.947), and YOLOv12 (0.92). To the best of our knowledge, this is the most extensive publicly available dataset of its kind, covering 25 categories of chemistry laboratory apparatuses and establishing a strong foundation for future research in laboratory automation.

## Background & Summary

In computer vision, object detection is a key task that enables automated systems to understand and interact with their environment^1,2^. Similarly, in chemical laboratory operations, apparatus detection is essential for efficient resource management and optimal equipment usage across various sectors. Many institutions struggle with equipment not being used enough because of neglect and mismanagement, so tracking and monitoring are important to make sure everything is used efficiently^3^. In areas like pharmaceutical analysis^4^, apparatus detection ensures the effective use of instruments, helping to develop practical skills and readiness for real-world applications^5^. In industrial research, detecting equipment functionality facilitates the integration of portable, flexible devices with existing setups, enhancing precision, cost-effectiveness, and efficiency without requiring extensive modifications^6,7^. Recently, object detection has become increasingly important in automating laboratory operations, particularly in the chemical sciences^8^. With the growth of deep learning methods, there is an increasing need for large, diverse, and high-quality datasets to train and evaluate these algorithms^9^. However, object detection in chemical laboratories presents unique challenges, such as dealing with transparent materials, overlapping equipment, and complex backgrounds^10^.

Various datasets have been proposed to support automated lab operations. For example, the CViG dataset was developed to detect chemistry glassware in real time and under varying lab conditions^11^. Another study introduced a dataset focused on improving lab safety by detecting personal protective equipment (PPE) compliance in real-time, reducing the need for manual safety checks^12^. A different dataset includes hands of experimenters interacting with the apparatus, which helps simulate actual lab working conditions more accurately^13^. Other studies have investigated the automatic identification of lab equipment to enhance safety and adaptability in changing lab environments^14^, analyzed the importance of accurate object localization for robotic systems^15^, and addressed the challenges of recognizing transparent vessels and overlapping tools^16^. Additional research has shown that using AI-driven vision systems with lab robots helps spot missing pipette tips and wrong liquid levels in real-time detection, making lab work more accurate and reliable^17^. Besides, another study analyzes how combining object detection with action recognition can provide a deeper understanding of lab tasks^18^. After that, object detection has been used to monitor safety risks and assist with automated experiment documentation^19^.

We present a dataset consisting of 4,599 images captured from actual laboratory environments. The dataset contains annotations for 25 commonly used chemical laboratory apparatuses, including Beaker, Conical Flask, Funnel, Glass Rod, Pipette, and so on. Unlike earlier datasets, our images cover a wide range of practical conditions such as occlusion, overlapping tools, varied lighting, different viewing angles, and partial visibility due to the complex environment shown in the figure. These characteristics make our dataset highly suitable for training deep learning models that need to perform well in non-ideal, real-world scenarios. The data are split into training, validation, and test sets to support robust model development and benchmarking. Our dataset directly addresses several challenges found in previous research studies:

- **Limited class diversity and real-world variability**: Many existing datasets focus on a few types of glassware or tools. All the related datasets are cited in Table 1. Our dataset introduces 25 different classes in varied environments to reflect real chemical lab usage.
- **Inadequate support for downstream lab applications**: Object detection plays a crucial role in enabling functions such as inventory tracking, automated experiment recording, and real-time safety alerts^20^. Our dataset is built to support these applications by providing diverse, annotated images that reflect realistic laboratory settings. Prior studies have emphasized the significance of identifying and localizing lab apparatus to improve robotic adaptability and enhance experimental safety^15^. Additionally, detecting apparatus accurately in complex environments has been shown to improve safety monitoring and experiment documentation^19^.

This work presents one of the most diverse and realistic public datasets of chemical laboratory equipment detection. It supports a wide range of research directions including equipment tracking, experiment documentation, lab safety, and human-object interaction modeling in scientific environments.

**Table 1:** Information of reviewed datasets.

Datasets	Number of Images	Number of Classes
^11^	4072	7
^12^	481	4
^13^	5078	7
^14^	2246	21
^15^	2246	21
^16^	2181	14
^18^	5078	7 (6 Apparatus and Hand)
**Our Dataset**	**4599**	**25**

## Methods

### Dataset creation: acquisition, annotation, and preprocessing workflow

#### Acquisition equipment

In developing a dataset for automated laboratory operations, incorporating images from devices with varying camera specifications is essential to ensure diverse data collection^21^. As detailed in Table 2, the use of cameras with different resolutions, focal lengths, and sensor qualities allows for the capture of distinct visual details of lab equipment and environments. This diversity enhances the dataset’s robustness, enabling models to generalize more effectively across a wide range of real-world setups and conditions.

**Table 2:** Specifications of the devices used to capture dataset images.

Device Model	Camera Specification	Features
Samsung Galaxy A05s	**Triple Rear Camera:** 50 MP, f/1.8 (wide), 1/2.8”, 0.64 μm, AF 2 MP, f/2.4 (macro) 2 MP, f/2.4 (depth)	LED flash
OnePlus 9R	**Quad Rear Camera:** 48 MP, f/1.7, 26 mm (wide), 1/2.0”, 0.8 μm, PDAF, OIS 16 MP, f/2.2, 14 mm, 123^°^ (ultrawide), 1/3.6”, 1.0 μm 5 MP, f/2.4 (macro) 2 MP, f/2.4 (monochrome)	Dual-LED flash, HDR
Samsung Galaxy S21	**Triple Rear Camera:** 12 MP, f/1.8, 26 mm (wide), 1/1.76”, 1.8 μm, dual-pixel PDAF, OIS 64 MP, f/2.0, 29 mm (telephoto), 1/1.72”, 0.8 μm, PDAF, OIS, 1.1x optical zoom, 3x hybrid zoom 12 MP, f/2.2, 13 mm, 120^°^ (ultrawide), 1/2.55”, 1.4 μm, Super Steady video	LED flash, auto-HDR, panorama
Huawei GR5	**Dual Rear Camera:** 12 MP, f/2.9 2 MP (depth)	Auto Flash, Face Detection, Touch to Focus

#### Data collection

Data for this study were gathered through fieldwork conducted in controlled laboratory settings at two locations in Dhaka, Bangladesh: the UIU Bio-Chemical Laboratory at United International University and Dhaka Imperial College. To capture a diverse range of image qualities and perspectives, four different smartphone cameras were used. This multi-device approach aimed to replicate the varying conditions under which chemical apparatuses may appear in real-world laboratory environments, enhancing the dataset’s realism and generalizability.

#### Dataset annotation and labeling

**Fig. 1:**
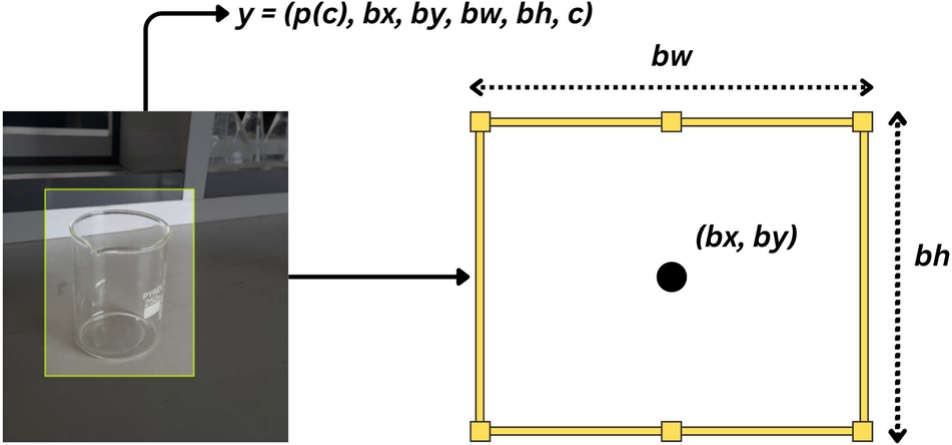
Image annotation with bounding box regression.

**Fig. 2:**
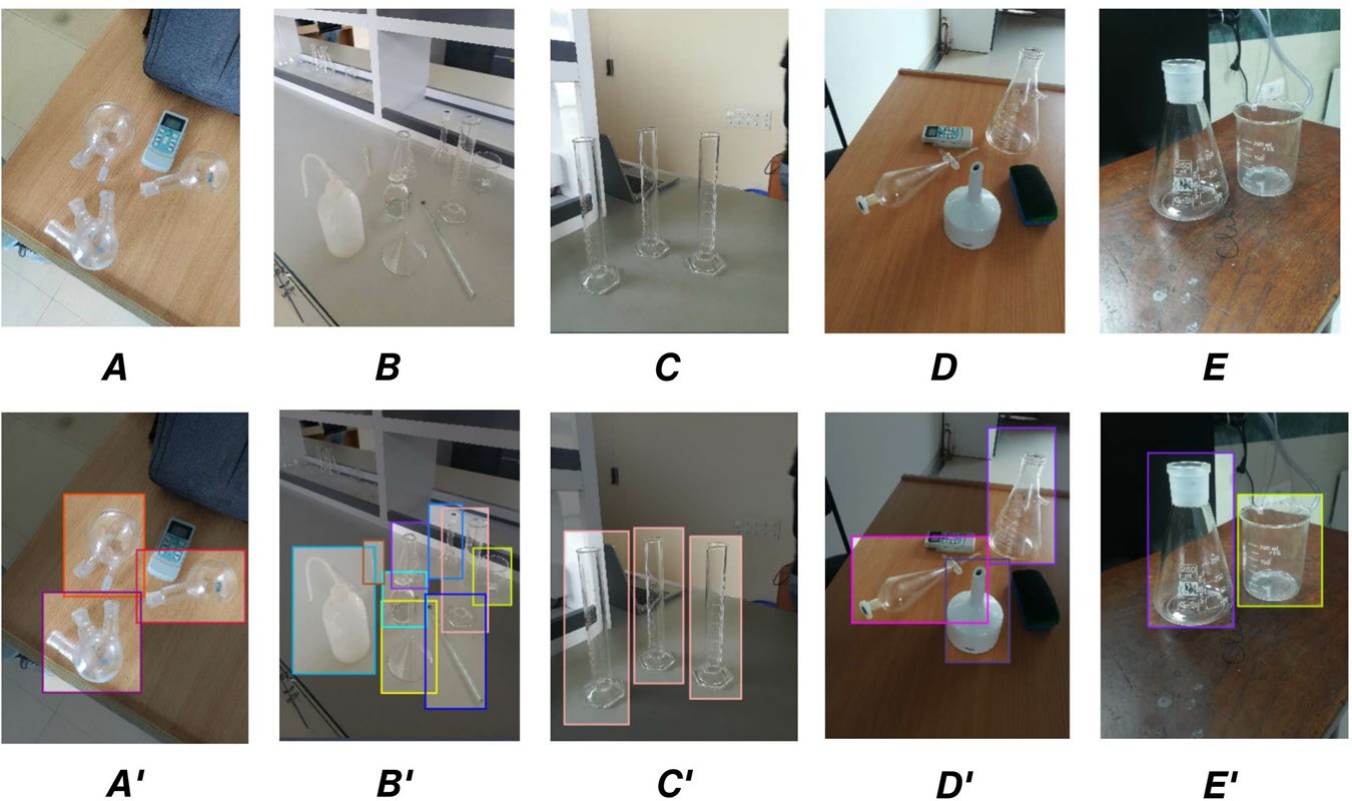
Visualization of bounding box annotations for lab apparatus.

The dataset was annotated using Roboflow, an online platform that streamlines image labeling for object detection tasks. We employed bounding box regression^22^ to annotate each image. This method involves drawing rectangular boxes around objects to highlight their presence within an image. Each bounding box is defined by four parameters: width (bw) and height (bh), representing the dimensions of the box; class (c), denoting the object category; and center coordinates (bx, by), indicating the box’s position within the image. Bounding box regression is a widely adopted technique in object detection, enabling precise localization and classification of objects. Figure 1 illustrates the bounding box regression concept, while Fig. 2 shows the annotation process using Roboflow. Each pair of images (A–E and A$^{\prime}$–E$^{\prime}$) shows the original lab setup and the corresponding annotated image with bounding boxes highlighting different types of laboratory equipment.

#### Data preprocessing

When creating the dataset version in Roboflow, some preprocessing steps were applied to maintain consistency and improve model accuracy. First, the “Auto-Orient” feature ensures that all images are correctly aligned by fixing any rotation issues caused by EXIF data. Then, the “Resize” step resizes all images to 640 × 640 pixels, making them uniform and easier for the model to process efficiently. These adjustments help models learn better and perform more accurately in complex and real-world scenarios.

### Experimental design

This experiment was designed to train object detection models for identifying chemical laboratory apparatus by first constructing a well-structured and diverse dataset. Images were captured across multiple locations to include a wide range of laboratory equipment and environmental conditions. To enhance robustness and generalizability, we varied lighting, backgrounds, and camera angles, enabling the models to learn object variations caused by real-world factors. This approach minimizes bias and improves model performance in practical applications.

After image collection, the files were stored in both local and cloud storage to ensure accessibility and secure backup. The dataset then underwent a data cleaning process, during which low-quality, blurry, or irrelevant images were removed to maintain a high standard for subsequent annotation and model training. Following cleaning, data annotation was conducted using Roboflow, where each object was accurately labeled with bounding boxes and corresponding class names. To ensure labeling consistency and precision, multiple annotators participated in the process. The annotated dataset was then refined in the data preparation phase, involving preprocessing techniques such as orientation correction, resizing, normalization, and contrast enhancement. Additionally, feature extraction was performed to emphasize key visual attributes critical for effective object detection.

To ensure effective model training, the dataset was randomly split into three subsets: 70% for training, 20% for validation, and 10% for testing. This distribution allowed the models to learn from a substantial portion of the data while reserving unseen samples to evaluate generalization performance. During the training phase, we configured hyperparameters, selected suitable neural network architectures, and trained the models using the training set. The validation set was used to fine-tune parameters and mitigate overfitting.

**Fig. 3:**
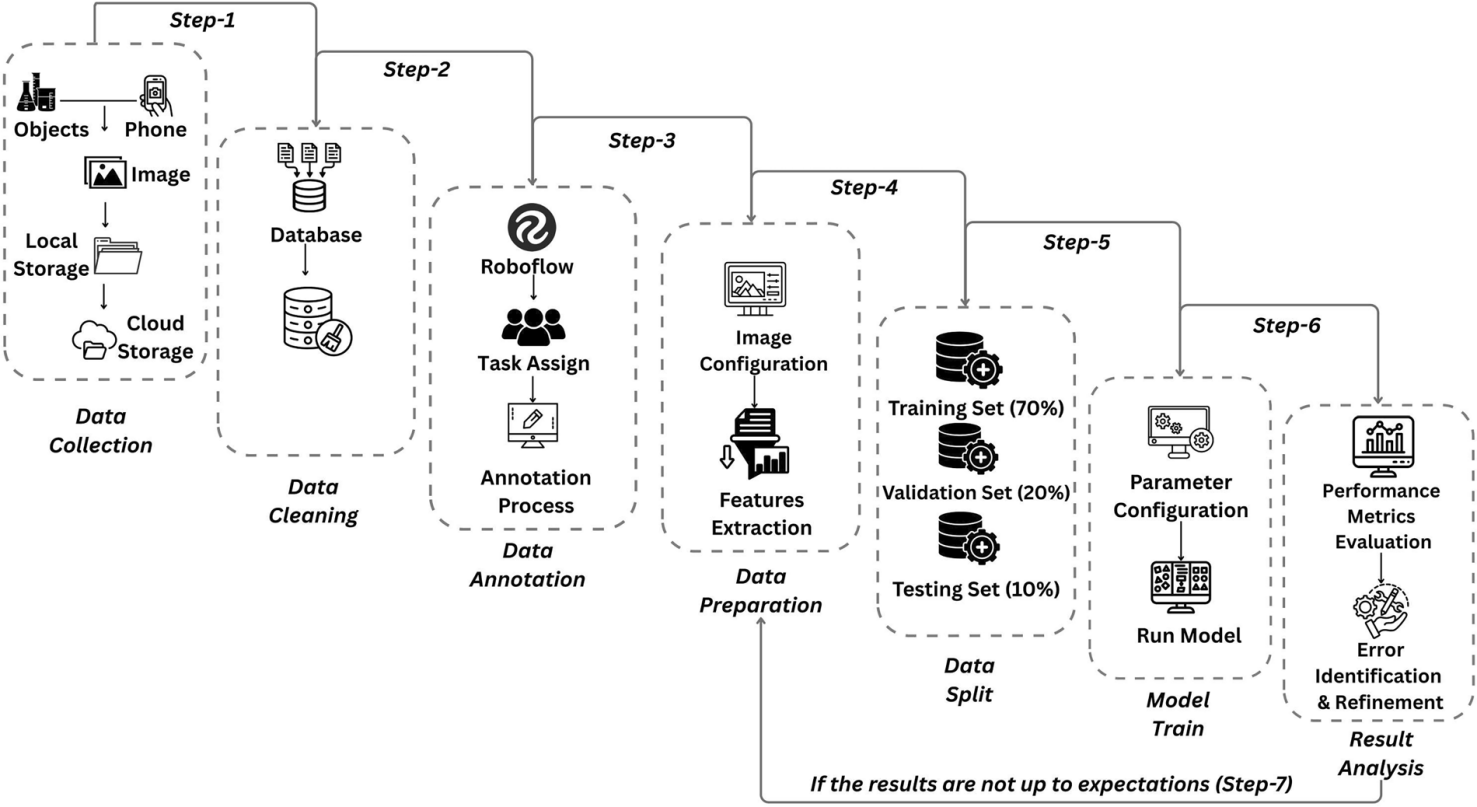
Framework of the experimental design pipeline.

In the evaluation phase, further detailed in the technical validation, we assessed model performance using metrics such as precision, recall, confusion matrix, and mAP@50. We also conducted error analysis by identifying misclassified instances, correcting annotation inconsistencies, and refining preprocessing steps. When performance fell short of expectations, iterative adjustments were made across the pipeline. The overall experimental framework is depicted in Fig. 3.

## Data Record

The dataset used in this study is freely available to everyone and can be accessed online through the Figshare platform^23^. This means that researchers, developers, and anyone interested can download and use the dataset for their experiments or analysis without any restrictions. Sharing the dataset openly helps promote transparency, reproducibility, and further research in the field. A detailed breakdown of the dataset structure and the type of information it contains is provided below.

### Dataset distribution analysis

The dataset comprises 4,599 images spanning 25 classes, with a total of 6,960 annotated instances. To support balanced and unbiased model training, we employed a random splitting technique to divide the dataset into three subsets: 70% (3,220 images) for training, 20% (920 images) for validation, and 10% (459 images) for testing. In this method, images are shuffled and distributed without regard to their original order or grouping^24^, minimizing the risk of bias that could arise from sorted or clustered data. This randomized approach ensures that each subset contains a representative mix of classes and conditions, thereby enhancing model generalization and performance. Images for each apparatus class were captured using all four smartphones to incorporate device-level variation in the dataset. This deliberate strategy helps reduce device-specific bias and introduces variation in lighting, resolution, and angle. The complete list of classes, their annotation counts, and the annotation distribution across devices is presented in Table 3.

**Table 3:** Device-wise distribution of per-class object annotations.

Serial	Class Name	Per ClassAnnotations	SamsungGalaxy A05s	OnePlus9R	SamsungGalaxy S21	HuaweiGR5
1	Beaker	395	97	107	90	101
2	Buchner_Funnel	247	63	57	70	57
3	Burette_Stands	302	74	70	83	75
4	Calorimeter	338	90	101	74	73
5	Conical_Flask	406	89	104	105	108
6	Funnel	231	67	61	49	54
7	Glass_Rod	398	85	94	108	111
8	Measuring_Cylinder	210	48	47	57	58
9	Mechanical_Balance_Scale	245	54	61	65	65
10	Nessler_Reagent_Bottle	245	65	65	64	51
11	Pipette	259	59	61	69	70
12	Porcelain_Mortar_Pestle	268	67	77	65	59
13	Precision_Weight_Scale	244	54	61	64	65
14	Reagent_Bottle	259	59	58	85	57
15	Round_Bottom_Flask_Borosilicate_Glass_1_Neck	362	87	71	101	103
16	Round_Bottom_Flask_Borosilicate_Glass_2_Neck	267	71	56	68	72
17	Round_Bottom_Flask_Borosilicate_Glass_3_Neck	263	64	60	72	67
18	Separating_Funnel	315	80	80	76	79
19	Spirit_Lamp	242	65	65	61	51
20	TestTube_Holder	256	79	69	52	56
21	Test_Tube	255	47	68	69	71
22	Volumetric_Flask	223	47	45	63	68
23	Volumetric_Pipet	280	76	70	59	70
24	Wash_Bottle	207	49	42	63	53
25	Weighing_Bottle	243	65	65	62	51

### Folder structure of the dataset

We created a custom dataset specially designed for our research on detecting and recognizing objects in chemical laboratories. The dataset folder includes important files like metadata.csv, which lists detailed information about each image, and data.yaml, which tells the model how to find and use the data.

**Fig. 4:**
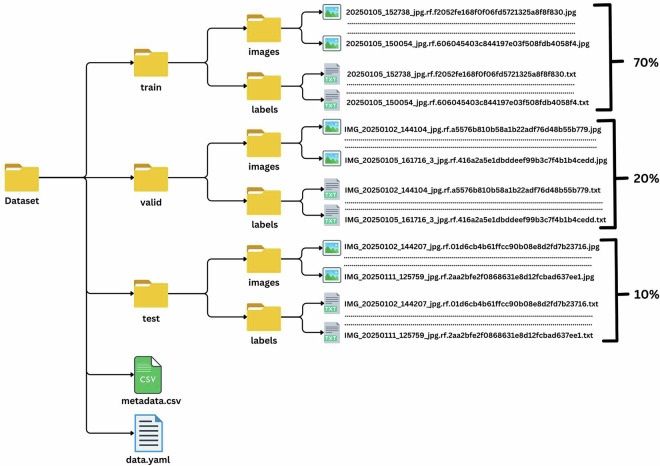
Folder structure of the dataset.

The dataset is divided into three main folders: train, valid, and test, to organize the data effectively. Each folder has two subfolders: images, which contain uniquely named pictures of chemical apparatus, and labels, which hold text files with annotations. These annotations provide essential details like class labels and bounding box coordinates for object detection. Figure 4 demonstrates the folder structure.

#### Metadata

The “metadata.csv” file, where relevant data concerning each image and corresponding annotations. There is one record for each image, including the name of the image used as an individual identifier for distinct images of chemical lab equipment. It also specifies the set type: train, valid, and test, which indicates the role of every image in the model-building process. For interpretability, every image is referenced against a Class_ID and a Class_Name that identify the object class identified within the image. The annotation also includes the X and Y coordinates and the Width and Height of the bounding box that define the exact position and size of the identified object in the image. In addition to that, a Device_Name is included to identify the target device on which each image was captured. The efficiently organized and structured nature of the metadata facilitates convenient data management and supports successful model training on object detection tasks. The fields included in “metadata.csv” are briefly described in Table 4.

**Table 4:** Metadata record for an annotated image.

Field	Values
Image	IMG_20250211_150120_jpg.rf.254a07754e8597e9fca021a326497c6.jpg
Set	train
Class_ID	2
Class_Name	Burette_Stands
X	0.48984375
Y	0.57109375
Width	0.5421875
Height	0.49296875
Device_Name	OnePlus 9R

## Technical Validation

### Evaluation matrics

In this study, several performance metrics are used to evaluate the object detection model. Precision is a performance metric that measures the proportion of correctly identified positive instances among all instances predicted as positive^25^. In object detection, it indicates the ratio of correctly predicted bounding boxes to the total number of predicted bounding boxes, reflecting the model’s accuracy in positive predictions (Equation 1). Recall measures the ability of the model to correctly identify all relevant objects within an image^26^. It is the ratio of correctly predicted positive instances to the total actual positives, indicating how well the model captures all target objects (Equation 2). Mean Average Precision (mAP) is a comprehensive evaluation metric used in object detection tasks to assess both precision and recall across different object classes^27^. It is calculated by first determining the Average Precision (AP) for each class, based on the area under the precision-recall curve, and then averaging the average precision scores over all classes (Equation 3). Additionally, the confusion matrix serves as a performance evaluation tool that summarizes classification outcomes by comparing the predicted outputs with the actual ground truth labels, indicating the numbers of True Positives, True Negatives, False Positives, and False Negatives^28^. In binary or multi-class classification, and object detection tasks involving classification, it provides a tabular representation in Table 5 of four key outcomes.

1 $$\,{\rm{Precision}}\,=\frac{TP}{TP+FP}$$

2 $$\,{\rm{Recall}}\,=\frac{TP}{TP+FN}$$

3 $$\,{\rm{mAP}}\,=\frac{1}{N}\mathop{\sum }\limits_{i=1}^{N}A{P}_{i}$$

Key terms and abbreviations used in the evaluation metrics are defined as follows:

**Table 5:** Structure of the confusion matrix for binary classification.

	Predicted Positive	Predicted Negative
**Actual Positive**	True Positive (TP)	False Negative (FN)
**Actual Negative**	False Positive (FP)	True Negative (TN)

- **TP (True Positives)**: Correctly detected objects.
- **TN (True Negatives)**: Correctly identified the absence of objects.
- **FP (False Positives)**: Incorrectly detected objects.
- **FN (False Negatives)**: Objects that were present in the image but not detected by the model.
- **N**: Total number of object classes.
- *AP*_*i*_ Average Precision for class *i*.

### Result analysis

Our dataset comprises images containing various laboratory objects captured using multiple smartphone cameras. Each image is annotated with bounding boxes and class labels to indicate the location and type of each object. To utilize this annotated data effectively for tasks such as automatic identification and localization of objects in new images, object detection models are essential. These models simultaneously classify and locate objects within an image^29^, making them critical for applications like inventory management, real-time recognition, augmented reality, robotics, and smart device integration. Table 6 presents the distribution of images across the training, validation, and testing subsets, ensuring a balanced split for reliable model evaluation.

**Table 6:** Distribution of images across dataset subsets.

Subset	Number of Images	Percentages
Training	3220	70%
Validation	920	20%
Testing	459	10%
**Total**	**4599**	**100%**

Among the most used object detection models are the different versions of the YOLO models^30^. YOLO models can process images fast and maintain high accuracy, making them well-suited for real-time object detection tasks, which makes the models valuable^31^. The RF-DETR model is also important because it uses advanced transformer-based architectures to detect objects with greater contextual understanding, particularly in cluttered or complex scenes. The flexibility of our dataset, exported in both YOLO and COCO formats, ensures it can be used to train a variety of object detection models, enabling broad applicability across research and real-world scenarios.

We evaluated our dataset using seven state-of-the-art object detection models, all achieving strong performance with mAP@50 scores exceeding 0.90. RF-DETR, a transformer-based model^32^, achieved the highest accuracy with an impressive mAP@50 of 0.992, while YOLOv11^33^ delivered the best results among all YOLO models with a score of 0.987. Other models, including YOLOv9^34^ (0.986), YOLOv5^35^ (0.985), YOLOv8^36^ (0.983), YOLOv7^37^ (0.947), and YOLOv12^38^ (0.92), also demonstrated solid results. Detailed performance metrics such as Precision, Recall, and Mean Average Precision (mAP)^39^ of the best two models are presented in Table 7.

**Table 7:** Evaluation of state-of-the-art object detection models on the dataset.

Model Name	Precision	Recall	mAP@50
YOLOv11	96.7%	97.7%	98.7%
RF-DETR	99.1%	79.9%	99.2%

**Fig. 5:**
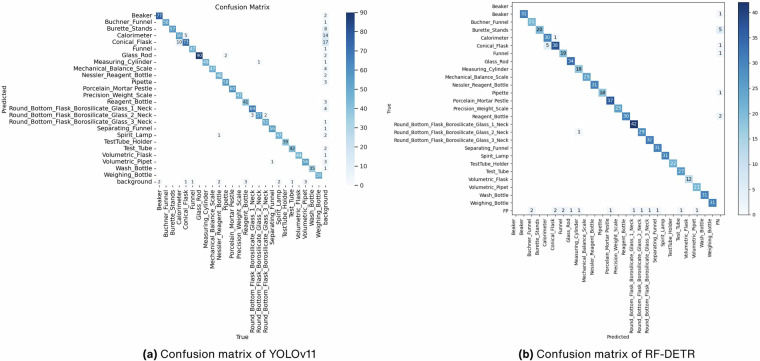
Confusion matrices of YOLOv11 and RF-DETR object detection models. (**a**) Confusion matrix of YOLOv11. (**b**) Confusion matrix of RF-DETR.

To visualize detection results, we also include confusion matrices of best two models^40^. These confusion matrices in Fig. 5 provide a detailed, class-by-class overview of how accurately the model classified 25 different laboratory objects. The diagonal cells show correct predictions for each class, while the off-diagonal cells highlight where the model confused one object with another. The accompanying color scale visualizes the frequency of correct and incorrect predictions, making it easy to spot patterns of misclassification. Overall, these matrices clearly illustrate the model’s strengths and weaknesses by comparing the true and predicted labels side by side.

**Fig. 6:**
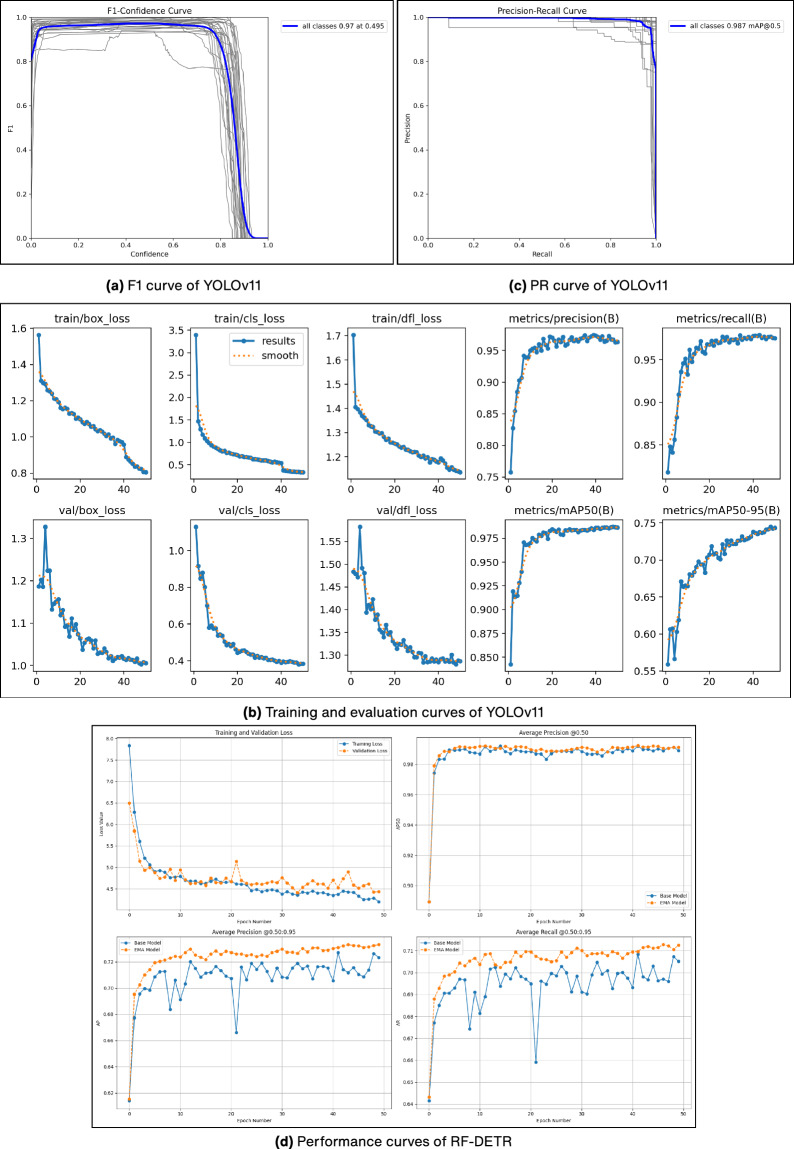
Performance analysis of YOLOv11 and RF-DETR object detection models. (**a**) F1 score versus confidence curve of YOLOv11. (**b**) Training and validation loss curves of YOLOv11. (**c**) Precision-recall curve of YOLOv11. (**d**) Training epoch evaluation metrics of RF-DETR.

In addition to the quantitative metrics, we attach model-wise visualizations such as F1-Score curves, Precision-Recall (PR) curves, and Result Matrices for each object detection model^41^. These plots provide deeper insights into the performance characteristics of each model across different confidence thresholds. Figure 6 presents these curves, offering a detailed view of how each model performs across differing confidence thresholds, including the top-performing models YOLOv11 and RT-DETR.

**Fig. 7:**
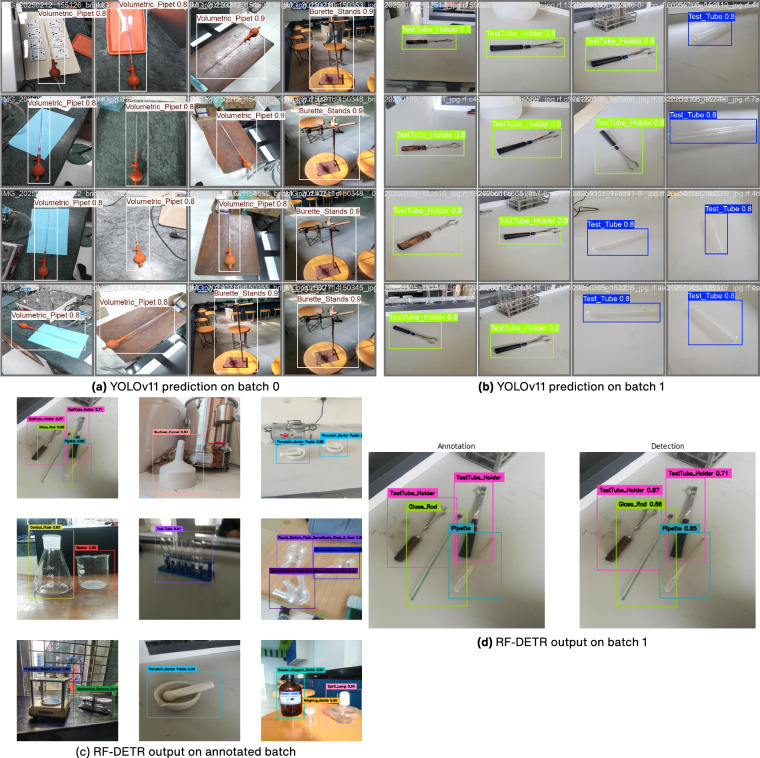
Object detection results of YOLOv11 and RF-DETR models. (**a**) YOLOv11 predictions on batch 0. (**b**) YOLOv11 predictions on batch 1. (**c**) RF-DETR output on annotated batch. (**d**) RF-DETR predictions vs annotations on batch 1.

Following the performance curves, we present visual examples of object detection results produced by each model. These Fig. 7 images showcase how each model identifies and localizes objects within various scenes from the test dataset.

In our research, the configuration parameters of the training environment are outlined in Table 8, and the hyperparameters used during the training process are listed in Table 9.

**Table 8:** System Configuration for Model Training.

Parameters	Setup
Graphics Processor	T4 GPU – YOLO Models
	A100 GPU – RF-DETR Model
Deep Learning Framework	PyTorch
CUDA Version	12.4

**Table 9:** Hyperparameters for training object detection models.

Parameters	Setup
Epochs	50
Batch-size	16
Image-size	640 × 640

## Usage Notes

The dataset follows the standard annotation format, so most of the object detection methods like the YOLO family methods (from YOLOv5 onward) and RF-DETR can readily use it. Each image is paired with a “.txt” annotation file containing class IDs and bounding box coordinates. Images are “.jpg” format and sorted into separate folders for training, validation, and testing. The dataset is freely available for researchers, institutions, and developers to use. Users are welcome to apply it to a wide range of different applications in laboratory settings. We also encourage users to convert the dataset into other formats (e.g., COCO (JSON), XML, CSV) using common open-source tools to suit their pipelines. Although no special software is required, we recommend using Python libraries such as OpenCV or Roboflow for previewing, pre-processing, or visualizing the data. We encourage users to share feedback, suggestions, and ideas, or to collaborate on future developments.

## Data Availability

The dataset was manually annotated using Roboflow (https://universe.roboflow.com/). Training used open-source object detection frameworks. YOLO models were implemented using Ultralytics codebase (https://github.com/ultralytics/ultralytics), and RF-DETR was trained using its official PyTorch repository (https://github.com/roboflow/rf-detr.git). We have shared all the code in a public GitHub repository: (https://github.com/SakhawatHossain/chem-lab-equipment-recognition.git). This includes scripts for training, evaluation, and visualizations, and simple guidance. Minor code modifications were made for compatibility, but no proprietary code was created. We suggest using a virtual environment to avoid dependency conflicts.
